# Psychotherapy as a Moderator of the Relationship Between Coping Strategies and Anxiety During the COVID-19 Pandemic

**DOI:** 10.3389/fpsyg.2021.764347

**Published:** 2021-10-18

**Authors:** Gianluca Merlo, Lucia Nicastro, Davide Taibi

**Affiliations:** ^1^Istituto per le Tecnologie Didattiche, Consiglio Nazionale delle Ricerche, Palermo, Italy; ^2^Società Italiana di Psicoterapia Funzionale, Naples, Italy

**Keywords:** COVID-19, coping strategies, anxiety, psychotherapy, mental health

## Abstract

The COVID-19 pandemic has led to the development of several risk factors for mental health, not only for people directly infected but also for those experiencing social isolation, loneliness, and concern for their health. The impact of these factors on individuals’ health and well-being also depends on the type of coping strategies adopted. Moreover, one of the main effects of COVID-19 is the increase in the prevalence of people who manifest anxious or depressive syndromes. This study investigates the relationship between coping strategies and a multidimensional assessment of anxiety symptoms measured during the COVID-19 pandemic in people who were having psychotherapy sessions, while participating in the study had psychotherapy sessions (clinical group) and those who have never done them (non-clinical group). The results of a questionnaire administered online to Italian subjects from June to July, 2020 were analyzed through multiple linear regressions methods to evaluate the role of psychotherapy as moderator between anxiety symptoms and coping strategies. The results of this study highlight that there are substantial differences in functioning between the clinical and non-clinical groups, underlying the key role of psychotherapy as a moderator between anxiety and the coping strategies of avoidance, social support, and positive attitude.

## Introduction

On the 11th March, 2020, the WHO defined the spread of severe acute respiratory syndrome coronavirus 2 (SARS-CoV-2) as pandemic. To date, millions of cases and deaths have been reported worldwide. Numerous national governments have implemented a range of virus containment measures, closing schools and workplaces, reducing travel, and canceling public events. The increase in social isolation, loneliness, concern for one’s health and that of loved ones, together with the concomitant economic crisis, are significant risk factors for the mental health of the world population (Holmes et al., 2020) not only for those who have directly contracted the infection but also for the entire population.

One of the main expected effects of COVID-19 is the increase in the prevalence of people who manifest anxious or depressive syndromes and who might engage in self-injurious behaviors (Mazza et al., 2020; Zhang et al., 2020; Wang et al., 2020b). Confirming the increased prevalence of symptoms related to anxiety, the prescription of anxiolytics in the United States of America increased by 34.1% from mid-February to mid-March, 2020 (Snow, 2020). The completion of the vaccination campaign cannot cancel these effects completely, as the effects of pandemics are long lasting. For example, symptoms related to post-traumatic stress disorders, anxiety, and depression in patients with SARS-CoV-1 have also been found more than 30months after the infection (Lam et al., 2009; Mak et al., 2009) and in healthcare personnel even 3years after the emergency (Ko et al., 2006). Pandemics are a source of stress that has repercussions on cognitive, emotional, physiological, and postural systems.

In this paper, we adopt the approach of the Functional Therapy (FT), an integrated body-mind therapy that considers the mentioned systems as deeply interconnected and congruent to each other (Rispoli, 2008; Perrella, 2017). According to this approach, assessing the anxiety of a person during the COVID-19 pandemic means taking into account negative emotions such as fear, anger, despair but also threatening thoughts about them or their loved ones, accompanied by a series of physiological phenomena such as muscle tension, tachycardia, abdominal discomfort, etc. Those who previously had limited experiences of anxiety and distress may experience an increase in the number and intensity of these episodes and some have developed psychopathological disorders (Brief UN Secretary-General Policy, 2020). Those who previously had pre-existing psychopathology may experience worsening of their condition and reduced functioning (Asmundson et al., 2020; Chen and Bonanno, 2020; Holmes et al., 2020). The severity of a stressor’s impact on an individual’s health also depends on the type of coping strategies used. The concept of coping and the strategies associated with it refers to the cognitive and behavioral efforts that help to reduce the pressure posed by a stressful situation (Folkman and Lazarus, 1985) and the perceived discrepancy between requests and resources (Compare et al., 2012; Martínez et al., 2020). Coping strategies can facilitate the management of stressful events and act as protective factors but they can also be dysfunctional and become risk factors for well-being.

Numerous studies have been carried out to evaluate which are the most effective and most deleterious coping strategies during the COVID-19 pandemic. A Chinese study carried out during the health emergency showed that subjects with a negative coping style have a higher level of psychological distress than those with a positive one (Wang et al., 2020a). A similar result is also found among subjects with a suspected COVID-19 infection, who spend more time searching for information about the disease, rarely using strategies aimed to cope with the stressor, and getting less social support (Yu et al., 2020). The best predictors of low levels of distress appear to be strategies of distraction, active coping, and seeking emotional social support (Park et al., 2020), hoping for the best, staying busy, humor (Kar et al., 2021), and the positive attitude toward life (Babore et al., 2020). A healthy and balanced diet, indulging in hobbies, being outdoors, and not reading news about the pandemic are also good predictors of low levels of anxiety and depression symptoms (Fullana et al., 2020). People with a higher level of education are those who show greater awareness and respect for prevention of the COVID-19 pandemic and adopt more proactive coping models, such as reading, physical activity, and seeking psychological support from the family (Fu et al., 2020). In disabled people, it was found that distraction and acceptance of the presence of the virus are the most frequently used strategies and are associated with the best results in terms of rehabilitation and quality of life (Umucu and Lee, 2020). Similar results can be found in children and more generally in students (Baloran, 2020; Orgilés et al., 2021). According to the mentioned literature, maladaptive coping is a risk factor for the development of psychopathological disorders or symptoms. This suggest that people who use negative coping strategies are those who are most likely to need access to psychotherapeutic treatments to improve their symptoms, quality of life, and coping style. Only a few studies investigated the relation between psychotherapy and coping styles (e.g., Kramer et al., 2010; Beutler et al., 2018; Starrs and Perry, 2018; Conceição et al., 2019) but to date no one has studied the psychotherapy as moderator between anxiety symptoms and coping styles. On the basis of this analysis, the present study aims to explore (a) what is the relationship between the coping strategies used during a stressful event, such as the COVID-19 widespread, and the anxiety symptoms measured taking into account four independent planes of the Self proposed by the Functional Therapy approach (cognitive-symbolic, emotional, physiological, and muscular) and (b) whether psychotherapy moderates the relationship between the multidimensional anxiety symptoms and coping strategies. The study compared in both cases a sample of clinical (people who were having psychotherapy sessions, while participating in the study) and non-clinical (people who have never had psychotherapy) subjects. In this paper, the term “psychotherapy session” indicates a face-to-face 60min individual meeting with a qualified psychotherapist aimed at treating mental health problems.

The results of this study highlight that there are substantial differences in functioning between the two groups, underlying the key role of psychotherapy as a moderator between anxiety and the coping strategies of avoidance, social support, and positive attitude.

## Materials and Methods

A questionnaire was administered online to Italian subjects from June to July, 2020. During this period many restrictions fell, such as the use of masks outdoors. Participants (general population aged 18years or older) were contacted through the authors’ network and posts on Facebook. To maximize the response rate of clinical subjects, authors asked psychotherapists and schools of psychotherapy to forward the questionnaire to their patients. This methodological choice introduces a possible bias that will be discussed later as limitation. Patients can be at different stages of therapy (in term of number of sessions done) and they may manifest different mental health problems. Moreover, psychotherapists can apply a broad range of different treatments based on different theoretical models. To reduce the bias related to the different phases of the therapy, authors defined an inclusion criterion of having done at least eight psychotherapy sessions according to Lambert (2013) who states that 50% of patients have reliable improvements by the 8th session of psychotherapy. About the comparability of different treatments, authors rely on the considerable number of meta-analyses that describe small and non-significant differences in therapeutic efficacy and effectiveness of the various form of psychotherapeutic interventions (Shapiro and Shapiro, 1982; Luborsky et al., 2002; Blatt and Zuroff, 2005; Wampold, 2013; Barth et al., 2016; Cuijpers, 2017).

### Procedure

The compiling instructions informed the participants that the answers were anonymous and that there were no right or wrong answers. Informed consent was obtained from all subjects involved in the study. A privacy policy at the beginning of the questionnaire described to the participants the purposes and methods of treatment operated by the data controller on the personal data collected. The research was submitted to the ethics committee of the University Polyclinic “Paolo Giaccone” of Palermo and obtained a favorable opinion for its development.

### Sociodemographic Questionnaire

The sociodemographic questionnaire included questions about gender, age, nationality, education, marital status, presence of children, and information about their experience with psychotherapy (whether they ever had it or not, frequency, number of sessions, and approach).

### Functional Anxiety Assessment Questionnaire

The functional anxiety assessment questionnaire (FQ) is a self-made questionnaire based on the psychotherapeutic functional theoretical model. It evaluated the manifestations of anxiety symptoms taking into account four different planes: cognitive-symbolic, emotional, physiological, and postural. The questionnaire consists of 12 items. For each item, subjects must express their degree of agreement in relation to a five-point Likert scale (1=never, 5=always). The questionnaire used for this study is available as Supplementary Material.

A Confirmative Factor Analysis was performed on all items corresponding to the four constructs measured as reported in Table 1. The final model scores well on the comparative fit index (CFI), Tucker – Lewis index (TLI), the Standardized Root Mean Square Residual (SRMR), and the root mean square error of approximation (RMSEA) equal to, 0.91, 0.88, 0.05, and 0.005, respectively.

**Table 1:** Functional anxiety assessment questionnaire (FQ) item saturation.

Latent factors	Item	SE	Z	std.all	*p*	sig
Cognitive-symbolic	FQ.1	0.00	NA	0.64	NA	NA
Cognitive-symbolic	FQ.2	0.14	6.91	0.63	0.00	***
Cognitive-symbolic	FQ.5	0.11	5.40	0.38	0.00	***
Emotional	FQ.3	0.00	NA	0.56	NA	NA
Emotional	FQ.4	0.16	5.39	0.39	0.00	***
Emotional	FQ.6	0.14	5.94	0.44	0.00	***
Physiological	FQ.7	0.00	NA	0.49	NA	NA
Physiological	FQ.8	0.17	5.60	0.47	0.00	***
Physiological	FQ.9	0.21	6.08	0.57	0.00	***
Postural	FQ.10	0.00	NA	0.59	NA	NA
Postural	FQ.11	0.10	5.11	0.36	0.00	***
Postural	FQ.12	0.20	6.47	0.71	0.00	***

The reliability analysis of the items of the final model by calculating the Cronbach alpha is acceptable (Cronbach’s Alpha=0.73). A completed detailed validity assessment of the FQ is actually under examination in a new study.

### The Coping Orientation to the Problems Experienced-New Italian Version-25

The coping orientation to the problems experienced-new Italian version-25 (COPE-NVI-25; Foà et al., 2015) is the reduced version of the COPE-NVI (Sica et al., 2008) developed to detect the coping strategies summarized by the authors in five categories: social support, avoidance, positive attitude, problem orientation, and transcendent orientation. The tool consists of 25 items that can be answered *via* a four-point Likert scale (1=I usually do not do this at all, 4=I usually do this a lot). The COPE-NVI-25 proved to be an instrument as valid as the original COPE, but easier to administer. Authors reported that Cronbach’s Alpha index is between 0.63 and 0.96.

### Participants

A number of 432 participants answered the questionnaire, the demographic characteristics are presented in detail in Table 1. The data was processed to remove underage subjects and responses attributable to so-called careless responses (Meade and Craig, 2012), i.e., responses that do not reflect real scores. In particular, the number and mean of consecutive identical responses and the intra-individual variability of response were evaluated. Starting with the detection of these suspected cases, responses in which the scores obtained were greater than 1.9 SDs from the mean were removed as well as subjects who do not comply with the criterion of having done at least eight sessions of psychotherapy. Following the data cleaning process, the number of valid responses was 389, of which 76 were males and 313 females, with an age between 19 and 79years old.

Demographic characteristics are presented in detail in Table 2. The average age of this group was 44.38years (SD=11.66). With regards to the qualification, 75.19% of the subjects declared that they have obtained a qualification equal to or higher than the first level degree. About 185 subjects (42.70%) had psychotherapy sessions at least once in their life. Of these 185, 81 (42.70%) declared that they were continuing the therapy sessions at the time of completing the questionnaire. The subjects declared to have carried out an average of 87.19 therapy sessions (SD=72.84), these sessions took place in most cases on a weekly (62.34%) or biweekly (25.97%) frequency.

**Table 2:** Group characteristics.

	Female (*N*=311)	Male (*N*=76)	Overall (*N*=387)
Age			
Mean (SD)	44.3 (11.5)	44.6 (12.5)	44.4 (11.7)
Median [Min, Max]	43.0 [20.0, 79.0]	43.0 [19.0, 79.0]	43.0 [19.0, 79.0]
**Nationality**			
Other	2 (0.6%)	1 (1.3%)	3 (0.8%)
Italian	309 (99.4%)	75 (98.7%)	384 (99.2%)
**Education level**			
Upper secondary education diploma	68 (21.9%)	25 (32.9%)	93 (24.0%)
Specialization diploma	48 (15.4%)	8 (10.5%)	56 (14.5%)
First level degree	53 (17.0%)	8 (10.5%)	61 (15.8%)
Master degree	99 (31.8%)	20 (26.3%)	119 (30.7%)
Middle school diploma	1 (0.3%)	2 (2.6%)	3 (0.8%)
First level university master	8 (2.6%)	2 (2.6%)	10 (2.6%)
Second level university master	15 (4.8%)	4 (5.3%)	19 (4.9%)
Title of PhD	19 (6.1%)	7 (9.2%)	26 (6.7%)
**Marital status**			
Unmarried maiden	117 (37.6%)	27 (35.5%)	144 (37.2%)
Married	147 (47.0%)	41 (53.9%)	188 (48.3%)
Divorced	33 (10.6%)	2 (2.6%)	35 (9.0%)
Civil partnership	11 (3.5%)	4 (5.3%)	15 (3.9%)
Widow/er	3 (1.0%)	2 (2.6%)	5 (1.3%)
**Have you ever had psychotherapy?**			
No	153 (49.2%)	49 (64.5%)	202 (52.2%)
Yes	158 (50.8%)	27 (35.5%)	185 (47.8%)
**Are you currently continuing your psychotherapy sessions?**			
No	91 (57.6%)	15 (55.5%)	106 (57.3%)
Yes	67 (42.4%)	12 (44.4%)	79 (42.7%)
**Approximately how many psychotherapy sessions have you had so far?**			
Mean (SD)	88.7 (72.1)	78.8 (79.6)	87.2 (72.8)
Median [Min, Max]	60.0 [15.0, 400]	55.0 [8.00, 270]	60.0 [8.00, 400]

## Results

### Multiple Regression

Multiple linear regressions were conducted to predict the scores obtained on the cognitive-symbolic, emotional, physiological, and postural dimensions of the FQ based on coping strategies measured by the COPE-NVI-25: positive attitude, avoidance, problem orientation, transcendent orientation, and social support. The regressions were conducted on both people who had psychotherapy sessions at least once in their life (clinical sample) and people who had not (non-clinical sample). Tables 3, 4 report the results of the regressions.

**Table 3:** Results of regression analysis in people who were having psychotherapy sessions, while participating in the study.

Predictors	Cognitive-symbolic			Emotional			Physiological			Postural			Estimates	*CI*	*p*	Estimates	*CI*	*p*	Estimates	*CI*	*p*	Estimates	*CI*	*p*
Intercept	5.50	1.71–9.30	0.005	5.19	0.20–10.19	0.042	6.83	2.70–10.96	0.002	11.14	5.84–16.43	<0.001
Positive attitude	−0.51	−1.51–0.50	0.320	−0.55	−1.87–0.78	0.412	−1.56	−2.66–−0.47	0.006	−2.03	−3.44–−0.63	0.005
Avoidance	1.33	0.47–2.18	0.003	0.72	−0.41–1.85	0.207	0.99	0.06–1.92	0.038	0.47	−0.73–1.66	0.437
Problem orientation	−0.65	−1.79–0.49	0.263	0.44	−1.06–1.94	0.561	0.30	−0.94–1.54	0.632	−0.11	−1.70–1.48	0.888
Transcendent orientation	0.28	−0.26–0.83	0.299	−0.42	−1.13–0.29	0.245	0.22	−0.37–0.81	0.453	0.56	−0.19–1.32	0.142
Social support	1.11	0.21–2.02	0.017	1.05	−0.14–2.25	0.082	0.42	−0.56–1.41	0.394	0.49	−0.78–1.75	0.446
Observations	79			79			79			79		
*R*^2^/*R*^2^ adjusted	0.226/0.173			0.088/0.025			0.170/0.113			0.164/0.106		

**Table 4:** Results of regression analysis in people who have never had psychotherapy.

Predictors		Cognitive-symbolic	Emotional	Physiological			Postural			Estimates	*CI*	*p*	Estimates	*CI*	*p*	Estimates	*CI*	*p*	Estimates	*CI*	*p*
Intercept	9.23	6.43–12.02	<0.001	8.39	5.30–11.47	<0.001	4.01	0.84–7.18	0.014	6.58	2.93–10.24	<0.001
Positive attitude	−0.92	−1.51–0.32	0.003	−0.52	−1.18–0.14	0.120	−0.34	−1.02–0.33	0.320	−0.46	−1.23–0.32	0.249
Avoidance	0.24	−0.34–0.83	0.412	−0.11	−0.76–0.54	0.732	0.49	−0.18–1.15	0.149	0.55	−0.22–1.31	0.160
Problem orientation	−0.08	−0.69–0.53	0.793	0.35	−0.32–1.03	0.303	−0.01	−0.70–0.68	0.984	0.29	−0.50–1.09	0.468
Transcendent orientation	0.01	−0.30–0.31	0.966	−0.03	−0.37–0.30	0.851	0.49	0.14–0.83	0.006	0.09	−0.31–0.48	0.671
Social support	0.21	−0.34–0.76	0.448	−0.01	−0.61–0.60	0.984	0.33	−0.29–0.95	0.301	0.15	−0.57–0.86	0.686
Observations	202			202			202			202		
*R*^2^/*R*^2^ adjusted	0.066/0.042			0.014/−0.011			0.060/0.036			0.021/−0.004		

#### Cognitive-Symbolic and Coping

The results of the regression on the clinical sample indicated that the two predictors explained 22.65% of the variance [*R*^2^=0.0.23, *F*(6, 73)=4.27, *p*<0.05]. It was found that avoidance predicts the scores obtained in the cognitive-symbolic (*β*=0.32, *p*<0.01), as well as social support did (*β*=0.26, *p*<0.01). These results are not replicated in the non-clinical sample. The result of the regression identifies just one predictor: the coping strategy of positive attitude toward life [*R*^2^=0.07, *F*(6, 196)=2.78, *p*<0.05; *β*=−0.23, *p*<0.01].

#### Emotional and Coping

In both the clinical and non-clinical samples, no predictors were found that significantly predict the results in the emotional dimension (*p*>0.05).

#### Physiological and Coping

In the clinical sample, two predictors explained 16.97% of the variance [*R*^2^=0.17, *F*(6, 73)=2.99, *p*<0.05]. Positive attitude significantly predicts physiological scores [*β*=−0.34, *p*<0.01], as well as avoidance (*β*=0.23, *p*<0.1). In the non-clinical sample, a significant regression equation was found [*R*^2^=0.06, F(6, 196)=2.51, *p*<0.05]. In this sample, the orientation to the transcendent significantly predicts physiological scores (*β*=0.08, *p*<0.01).

#### Postural and Coping

The positive attitude toward life events significantly predicts the scores obtained on the postural dimension (*β*=−2.03, *p*<0.01). High scores in positive attitude are combined with low scores in the postural dimension. This predictor explains the 16.36% of the variance [*R*^2^=0.16, F(6, 73)=2.86, *p*<0.05].

### Moderation Analysis

Starting from the results of the multiple regression analysis, a hierarchical multiple regression analysis was conducted to test the hypothesis that the psychotherapy moderates the relationship between the distress on:

- The cognitive-symbolic dimension and the coping strategy of avoidance.
- The cognitive-symbolic dimension and the coping strategy of social support.
- The postural dimension and the coping strategy of positive attitude.

The scores on the FQ dimensions were indicated as dependent variable, while the coping strategies and having had psychotherapy or not (−1 no psychotherapy, 1 psychotherapy) were modeled as predictors. The independent variable and predictors were introduced in step 1, the interaction between psychotherapy and coping strategies in step 2. To avoid the multicollinearity, the residual centering technique (Lance, 1988; Little et al., 2006; Geldhof et al., 2013) was applied with the support of the R pequod package (Mirisola and Seta, 2016).

#### Cognitive-Symbolic and Avoidance

**Figure 1:**
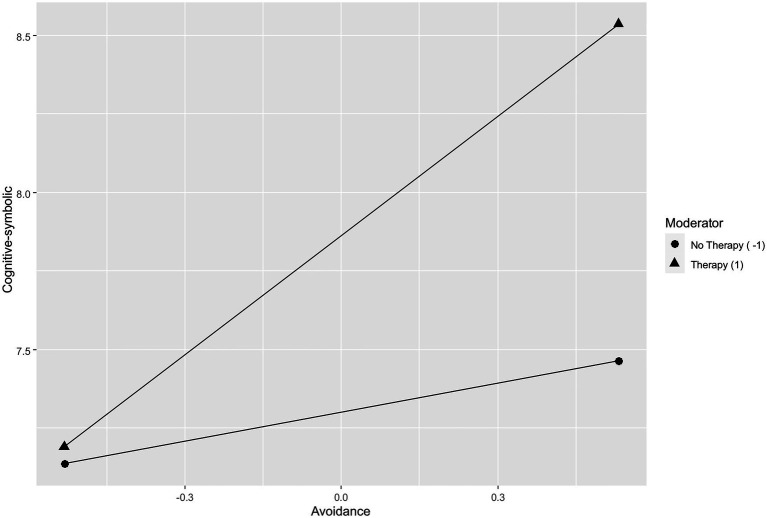
Simple slope showing the role of psychotherapy as moderator in the relationship between cognitive-symbolic and avoidance.

Positive attitude (*β*=−0.14, *p*≤0.05), avoidance (*β*=0.18, *p*≤0.01), and social support (*β*=0.16, *p*≤0.01) were found to be significant predictors of cognitive-symbolic scores. The interaction between psychotherapy and avoidance was significant (*β*=0.11, *p*≤0.05), indicating that whether or not had done psychotherapy moderates the effect of avoidance on the cognitive-symbolic. In particular, as shown in Figure 1, in the clinical sample, the more the subjects used the avoidance coping strategy, the more they obtained high cognitive-symbolic scores.

#### Cognitive-Symbolic and Social Support

**Figure 2:**
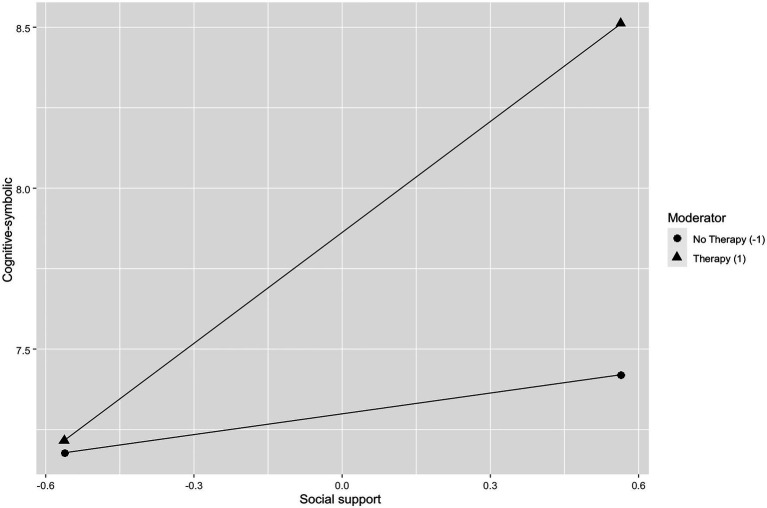
Simple slope showing the role of psychotherapy as moderator in the relationship between cognitive-symbolic and social support.

Positive attitude (*β*=−0.14, *p*≤0.01), avoidance (*β*=0.18, *p*≤0.001), and social support (*β*=0.16, *p*≤0.001) were predictors significative of the cognitive-symbolic. As shown in Figure 2, the more subjects use a social support-based coping strategy the more they scored significantly higher on the cognitive-symbolic dimension when if they had psychotherapy (*β*=0.11, *p*≤0.05).

#### Postural and Positive Attitude

**Figure 3:**
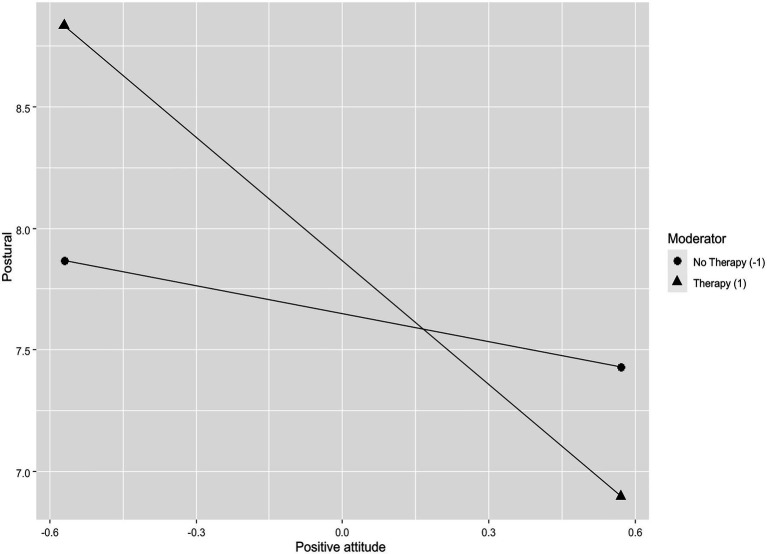
Simple slope showing the role of psychotherapy as moderator in the relationship between postural and positive attitude.

Positive attitude (*β*=−0.20, *p*≤0.001) and avoidance (*β*=0.13, *p*≤0.05) were significant predictors of postural. The interaction between positive attitude and therapy was significant (*β*=−0.13, *p*≤0.05). The more subjects used the positive attitude coping strategy, the more they get low scores on the postural dimension when if they had psychotherapy (Figure 3).

## Discussion

The consequences of coronavirus infections are pervasive and simultaneously affect children, adolescents, adults, and the elderly. This situation is compromising the possibility of developing one’s skills, knowledge, and potential to the fullest as basis for well-being and for living a full and satisfying life. The pandemic is jeopardizing not only health, economic stability, educational processes, but also planning, sharing, discovery, pleasure, calm, and the perception that the world and people are sources of nourishment and not of danger. The experience gained with other pandemics teaches us that the consequences of COVID-19 will not be resolved with the inoculation of vaccines but will persist for a medium-long time and will have a high social cost. Mental health is therefore called to an additional effort, experts must be able to intervene proactively, reducing as much as possible the conditions that create discomfort, and reactively intercepting promptly the signs of suffering, sometimes well hidden, promoting actions that can offer opportunities for growth, reinforcement, and rebalancing of personal resources. In addition to mental health experts, policy makers have to recognize and address the unprecedented social challenges presented by the COVID-19 pandemic. Many studies underline that social interventions aimed to promote the sense of community, social cohesion, social support, and social participation are important predictors of human health and well-being (e.g., Berkman and Glass, 2000; Chavis and Wandersman, 2002; Letcher and Perlow, 2009; Mak et al., 2009) also for multicultural societies and ethnic minorities (Wang and Ramsden, 2018; Wang and Morav, 2021).

In this context, this study is intended to provide a snapshot of the consequences of COVID-19 on the mental health of a sample of Italian subjects in order to reveal some risk and protection factors. The following paragraphs describe the main results obtained in the study and its main limitations.

### Main Results of the Study

The study explored the relationship between coping strategies and a multidimensional assessment of anxiety symptoms measured during the COVID-19 pandemic in people who were having psychotherapy sessions, while participating in the study and those who have never had them. Moreover, the role of psychotherapy as moderator between anxiety symptoms and coping strategies has been evaluated. Research results show that there is a substantial difference in functioning between patients in psychotherapy and those who have never had it. As predicted, psychotherapy moderates the relationship between multidimensional assessment of the anxiety and coping strategies. In particular, the moderation of psychotherapy influences the relationship between the cognitive level and social support, between the cognitive level and avoidance, and, finally, between the muscular one and the positive attitude toward life. In the first two cases, the subjects in the clinical sample who use the coping strategies of social support and avoidance the most obtain high scores on the cognitive dimension for the presence of negative fantasies, confusion, difficulty making decisions, rumination, and reduced planning. Social support can therefore also be a risk factor if the subject’s network of relationships causes stress, for example, discouraging the expression of emotions, making critical observations, or not providing the promised help (Lincoln, 2000). This result appears, at first sight, dissonant with a long tradition of research (e.g., Blazer, 1982; Cohen, 1988; House et al., 1988; Taylor, 2011), which has highlighted the positive aspects of social support, highlighting the positive consequences from the point of view of the health of the subjects who receive it, but it is consistent with what was found by Babore et al. (2020) on health professionals.

The risk associated with the avoidance coping strategy is known and consistent with the scientific literature. Avoidance, described as a person’s tiring attempt to deny, minimize, and evade direct confrontation with stressful requests, is generally linked to anxiety and depression (Cronkite and Moos, 1995; Blalock and Joiner, 2000; Penley et al., 2002; Marchand and Hock, 2003). Some authors (Holahan et al., 2005) have shown that the avoidance coping strategy can play a key role in generating a wide range of additional stressors. For example, avoidance can lead to financial or health problems, or aggravate tensions at work or within the family. Especially among women, hypertrophic rumination is associated with more severe and long-lasting depressive symptoms (Nolen-Hoeksema, 1998).

The third and final analysis of moderation that has obtained significant results relates to subjects who use the coping strategy of a positive attitude toward life. In these subjects, if belonging to the clinical sample, the higher their level of positive attitude, the more their perception of the rigidity of their posture, limbs, neck, and back muscles decreases. It is known that high levels of positive attitude are positively correlated with almost all dimensions of psychological well-being (Sagone and De Caroli, 2014) and also have a positive impact on the salary received (Mohanty, 2009a,b). This result is, once again, consistent with the study of Babore et al. (2020) carried out during the first peak of the pandemic in Italy on healthcare workers, in which the higher positive attitude of the subjects is, the lower is their perceived stress. Promoting a positive attitude toward life is therefore configured as a powerful protective factor from stress and malaise.

An additional result of the study is the identification of the orientation to the transcendent as predictor of physiological scores in the non-clinical sample. Currently, it is known that rigid adherence to religious beliefs can interfere with adaptive coping. The worst score obtained to the physiological dimension can be related, for example, to an external locus of control, delegating a legitimate healthcare to the will of a supernatural being (Carone and Barone, 2001).

### Limitations

The results of this study are promising but there are several limitations to mention that may jeopardize both internal and external validity. The sample involved in the study cannot be considered representative of the Italian people who have access to psychotherapeutic services. Considering that the percentage of people who have had psychotherapy sessions in Italy is relatively small (Vecchia, 2020), authors decided to use a convenience sampling, asking psychotherapists and schools of psychotherapy to involve their patients in the study. As a consequence, the number of people who have experience of psychotherapy is over-represented and this introduces a selection bias that limits the generalizability of the findings. The patients involved in the study had a different number of psychotherapy sessions and the reasons they asked for help are very heterogeneous. Although, an inclusion criterion of having done at least eight psychotherapy sessions (according to Lambert, 2013) has been established, the uneven composition of the sample can introduce a confounding effect that may influence the outcomes. For example, being in different phases of the therapy can influence the use of coping strategies (Kramer et al., 2010; Starrs and Perry, 2018; Conceição et al., 2019).

Moreover, considering that the psychotherapists of the patients involved in the study use many different approaches, multiple interventions are carried out to face the broad range of mental problems mentioned above. Authors relied on the functional equivalence of the various form of treatment according to many meta-analyses reporting a lack of significant differences among psychotherapeutic treatments (e.g., Shapiro and Shapiro, 1982; Blatt and Zuroff, 2005; Wampold, 2013; Barth et al., 2016; Cuijpers, 2017). This functional equivalence is called Dodo bird effect (Luborsky et al., 2002), a label inspired by Alice in Wonderland in which a Dodo states that “Everyone has won, and all must have prizes.” However, although, the studies reporting small and non-significant differences between treatments are very numerous, the Dodo bird effect is still controversial (Budd and Hughes, 2009). For example, meta-analyses have to take into account the role of the therapist’s allegiance that is highly predictive of the efficacy of interventions (Luborsky et al., 1999; Wampold, 2013). In addition, researchers highlight that the use of randomized control trials to identify specific effects of psychological therapy should be questioned in favor of systematic single case designs (Hallam, 2015). According to the considerations outlined above, a replication study should consider the interaction between the independent variables “diagnosis” and “type of treatment.”

With regards to the measure used in the study, the validity of the FQ must be improved for example accompanying it with another instrument or predictor. Therefore, although the confirmatory factor analysis of the FQ is acceptable, the questionnaire requires the application of sophisticated methodologies both quantitative (Kunnan, 1998) and qualitative (Banerjee and Luoma, 1997) in order to be validated.

Finally, the lack of longitudinal data cannot capture changes in psychological distress during both the psychotherapeutic treatment and the course of the COVID-19.

## Conclusion

The results of this study showed that psychotherapy can play a key role as moderator between anxiety symptoms and coping strategies. The detrimental effect of avoidance and negative social support coping strategies was lower in people who were having psychotherapy sessions at the time they participated in the study, while the protective action of the positive attitude in subjects in psychotherapy was significantly stronger.

## Data Availability Statement

The datasets presented in this study can be found in online repositories. The names of the repository/repositories and accession number(s) can be found at: https://doi.org/10.6084/m9.figshare.16742443.v1.

## Ethics Statement

The study was conducted according to the guidelines of the Declaration of Helsinki, and approved by the Ethics Committee of Azienda Ospedaliera Universitaria Policlinico Paolo Giaccone of Palermo, Italy (record number 6/2020 issued June 24, 2020). The patients/participants provided their written informed consent to participate in this study.

## Author Contributions

GM, DT, and LC: conceptualization, investigation, and writing—review and editing. GM: formal analysis, methodology, and writing—original draft preparation. All authors contributed to the article and approved the submitted version.

## Conflict of Interest

The authors declare that the research was conducted in the absence of any commercial or financial relationships that could be construed as a potential conflict of interest.

## Publisher’s Note

All claims expressed in this article are solely those of the authors and do not necessarily represent those of their affiliated organizations, or those of the publisher, the editors and the reviewers. Any product that may be evaluated in this article, or claim that may be made by its manufacturer, is not guaranteed or endorsed by the publisher.
