# Supplementary material for: Psychotherapy as a Moderator of the Relationship Between Coping Strategies and Anxiety During the COVID-19 Pandemic
Source: Front Psychol. 2021 Oct 18;12:764347. doi: 10.3389/fpsyg.2021.764347 (PMC8559725; doi:10.3389/fpsyg.2021.764347)
Supplement: Supplementary file 1 [file Table_1.DOCX]

Supplementary Material

# QUESTIONARIO FUNZIONALE DELL’ANSIA

Da quando è iniziata l’emergenza COVID-19, con quale frequenza si sono verificati i seguenti

fenomeni?

Modalità di risposta

0 = Mai; 1 = Raramente; 2 = Qualche volta; 3 = Spesso; 4 = Sempre

| Ho fantasie negative su ciò che potrebbe accadere | 0 | 1 | 2 | 3 | 4 |
| --- | --- | --- | --- | --- | --- |
| Penso che potrebbe accadere qualcosa di spiacevole | 0 | 1 | 2 | 3 | 4 |
| Mi riesce difficile prendere decisioni | 0 | 1 | 2 | 3 | 4 |
| Mi sento in uno stato di agitazione | 0 | 1 | 2 | 3 | 4 |
| Sono fiducioso rispetto a ciò che può accadere in futuro | 0 | 1 | 2 | 3 | 4 |
| Mi sento irritabile | 0 | 1 | 2 | 3 | 4 |
| Avverto sensazioni quali brividi, vampate di calore, formicolii o torpore | 0 | 1 | 2 | 3 | 4 |
| Ho la sensazione che mi manchi l'aria o di respirare a fatica | 0 | 1 | 2 | 3 | 4 |
| Avverto uno o più dei seguenti fenomeni: aumento della sudorazione, tachicardia, disturbi addominali, aumento della temperatura corporea, insonnia | 0 | 1 | 2 | 3 | 4 |
| Sento che la mia postura è rigida | 0 | 1 | 2 | 3 | 4 |
| I miei movimenti sono fluidi | 0 | 1 | 2 | 3 | 4 |
| Sento tensione muscolare negli arti, nei muscoli del collo, o della schiena | 0 | 1 | 2 | 3 | 4 |
